# Bone Cement Implantation Syndrome: A Report of Four Cases

**Published:** 2009-04

**Authors:** Pradeep Govil, P N Kakar, Deep Arora, Shibani Das, Nishkarsh Gupta, Deepak Govil, Sachin Gupta, Ashima Malohtra

**Affiliations:** 1 Consultant, Department of Anaesthesiology and Pain Management, Max Super specialty Hospital, Saket, New Delhi. 110017, India; 2 Director, Department of Anaesthesiology and Pain Management, Max Super specialty Hospital, Saket, New Delhi. 110017, India; 3 Senior consultant, Department of Anaesthesiology and Pain Management, Max Super specialty Hospital, Saket, New Delhi. 110017, India; 4 Senior consultant, Department of Anaesthesiology and Pain Management, Max Super specialty Hospital, Saket, New Delhi. 110017, India; 5 Attending consultant, Department of Anaesthesiology and Pain Management, Max Super specialty Hospital, Saket, New Delhi. 110017, India; 6 Consultant, Department of Anaesthesiology and Pain Management, Max Super specialty Hospital, Saket, New Delhi. 110017, India; 7 Senior resident, Department of Anaesthesiology and Pain Management, Max Super specialty Hospital, Saket, New Delhi. 110017, India; 8 Attending consultant, Department of Anaesthesiology and Pain Management, Max Super specialty Hospital, Saket, New Delhi. 110017, India

**Keywords:** Bone cement, Complications, Joint replacement surgeries

## Abstract

**Summary:**

Cardiovascular collapse following use of methylmethacrylate for lower limb surgeries has been reported. However there are no reports of cement reaction following shoulder arthroplasty. We report series of four patients exhibiting cement reaction. Two of our patients had cardiovascular collapse following cement insertion during hip arthroplasty. Severe hemodynamic derangement and transient hypoxemia was observed during cemented arthroplasty of shoulder and knee respectively. Peripheral vasodilatory effects of the cement monomer, fat and marrow embolism and activation of the clotting cascade in the lungs, all contribute to cement reaction. Early and aggressive resuscitation with use of vasopressors, establishment of invasive hemodynamic monitoring and surgical modifications are the key to prevention of catastrophic outcome.

## Introduction

Methylmethacrylate when used as cementing agent in arthroplasty has been associated with hypotension, hypoxia^1^–^3^ and intraoperative cardiac arrest in 0.6 to 1.0% patients^4^,^5^. The possible association between use of methylmethacrylate and cardiac arrest is unproven and more recently the importance of intraoperative fat embolization during cement insertion has been high-lighted^2^,^4^,^6^–^7^. Peripheral vasodilatation effect of the cement monomer, fat and marrow embolism, and activation of the clotting cascade in the lungs, all contribute to syndrome complex^8^,^9^ called as bone cement implantation syndrome (BCIS).

Hazards of using methylmethacrylate for fixation of components of a total hip arthroplasty^10^–^13^ and during total knee replacement with the use of devices for intramedullary alignment or with stemmed components have been reported^14^–^16^.

During review of literature we did not come across cement reaction following shoulder arthroplasty.

We are presenting four case reports of cement reaction in patients exhibiting symptomatology of different severity undergoing cemented arthroplasty. This report provides further clinical evidence of the risks and consequences of fat embolization and strategies to minimize morbidity and mortality.

## Case 1

A 70-year-old female presented to the operating room with intertrochanteric fracture right femur for total hip replacement. She had history of occasional dyspnea and hypertension managed with amlodipine 5mg OD. Preoperative biochemical and hematological profile was within acceptable limits. Electrocardiogram was unremarkable and dobutamine stress echocardiogram was negative for provocable ischemia with normal ventricular size, functions and left ventricular ejection fraction of 65%. Surgery was conducted in combined spinal epidural anaesthesia(CSE) in left lateral position. Patient's hemodynamic parameters were stable following CSE.

Liquid methylmethacrylate (20gm) was injected into the medullary canal using cement gun and long stem femoral prosthesis was inserted. Approximately 4 minutes after insertion of the stem the patient had uprolling of eye-balls. Patient's peripheral pulses disappeared and blood pressure was not recordable. ECG monitor showed arrhythmias with electromechanical dissociation. Endotracheal intubation was done and cardiopulmonary resuscitation was started after turning the patient supine. 5ml epinephrine (1:10000) was given intravenous and repeated at regular intervals (total 25ml).

After 25minutes of external cardiac massage, infusion of crystalloid and vasopressors, spontaneous cardiac rhythm and satisfactory blood pressure could be achieved. Patient was on epinephrine (0.2mcg.kg^−1^ min^−1^) and norepinephrine (0.3mcg.kg^−1^ min^−1^) infusion. An arterial line and subclavian central venous line was inserted. The wound was closed rapidly and patient was transferred to the intensive care unit (ICU).

On arrival to ICU patient was unconscious, orotracheally intubated, pulse 130beats.min^−1^, BP 90/40 mmHg on epinephrine and norepinephrine infusion and SpO_2_ 97% oxygen on artificial ventilatory support with FiO_2_ 1.0. Vasopressin infusion (0.03 IU.min^−1^) was added later to maintain mean blood pressure above 65mmHg.

Postoperative ECG was normal and echocardiography showed good L.V. function (LVEF 65%) with no regional wall motion abnormality and mildly raised pulmonary artery systolic pressure. Troponin T test was negative and cardiac enzymes were normal. Patient became conscious after six hours and had to be sedated using midazolam infusion 1mg.hr^−1^. Urine output decreased after 12 hours and injection frusemide infusion (20mg.hr^−1^) was started. Serial arterial blood gases after the cardiac arrest showed progressive metabolic acidosis, despite excellent oxygenation. Sodium bicarbonate infusion (20meq.hr^−1^) was added and increased as per requirement.

After prolonged efforts of resuscitation for 22 hours, patient had pulseless ventricular tachycardia which could not be reverted and she died.

## Case 2

A 76-year-old woman presented for total hip replacement of right side. She had previously sustained a right intertrochanteric fracture, which had been repaired with a dynamic hip screw with plate. She had previous history of hypertension and burns with multiple contractures and scars. The preoperative biochemical and hematological profile, electrocardiogram and echocardiogram were within acceptable limits. Right internal jugular vein was cannulated and combined spinal epidural anaesthesia was given. Patient was hemo-dynamically stable with acceptable SpO_2_ and EtCO_2_ before starting surgery. Surgery was performed in left lateral position.

No systemic effects were noted after fixation of acetabular component with cement. Four minutes after injection of liquid methylmethacrylate (20gm) into the intramedullary canal with an injection gun and fixation of long stem femoral prosthesis, systolic blood pressure decreased to 40mmHg, patient had bradycardia (heart rate 30/min) with atrial and ventricular ectopics with respiratory arrest. Orotracheal intubation was performed and lungs were ventilated using 100% oxygen after turning patient supine. Atropine 1.2mg and mephentermine 15mg was given intravenously followed by epinephrine in 5mcg increments. Sinus rhythm returned and decreased blood pressure was treated using crystalloids and colloids and norepinephrine infusion (0.2 mcg.kg.^−1^min^−1^). ABG analysis revealed metabolic acidosis and after correction of acid base status, patient's spontaneous effort and reflexes came back. In next 30 minutes patient became conscious. When all vital parameters were found normal norepinephrine infusion was tapered off and extubation was done. Patient was transferred to the ICU of observation. Post-operative hematological and biochemical parameters were found to be normal. Subsequently she had smooth recovery and was transferred to the ward next day.

## Case 3

A 74-year-old woman with previous history of hypertension presented to the hospital with secondary osteoarthritis of right shoulder and surgeons planned total shoulder replacement. Her preoperative biochemical and hematological profile was within normal limits. Surgery was done under general anaesthesia with tracheal intubation and controlled ventilation. Pulse, ECG, SpO_2_, EtCO_2_ were monitored. Patient was kept in sitting position for surgery. After insertion of methylmethacrylate and cemented implant, blood pressure decreased (BP 59/43mmHg), EtCO_2_ decreased to 4mmHg and SpO_2_ dropped to 60%. Cardiovascular examination did not reveal any murmur. Patient was turned to the supine position and was successfully resuscitated with 100% oxygen; intravenous administration of fluids, mephentermine (in 10mg increments, i.v., total dose 30mg) and epinephrine 5mcg. Reversal from anaesthesia and postoperative course was uneventful.

## Case 4

A 74-year-old woman with no history of medical problems presented to the hospital with secondary osteoarthritis left knee. She was posted for total knee replacement (TKR). Surgery was done under combined spinal epidural anaesthesia in supine position. Tourniquet was used during surgery which took 64 minutes. At the time of deflation of tourniquet, SpO_2_ decreased to 65%. Blood pressure and pulse remained stable and consciousness was not obtunded. 100% oxygen was administered using anaesthesia machine and it took 4 minutes for the SpO_2_ to increase above 90%. Postoperatively patient remained confused for 6 hours and required prolonged oxygen therapy for 48 hours. Serial ABG's were normal excepting increased alvelo-arterial oxygen difference.

In all four cases fluid management was done as per perioperative fluid therapy guidelines.

## Discussion

Transient hypotension occurs in approximately one-third of patients during cemented hip arthroplasty^5^,^6^.

Case 1 had BCIS with fatal outcome 22 hours after initial cardiovascular collapse. This patient was hemodynamically stable although on inotropic support. Postoperative ECG and echocardiogram did not show any change. Metabolic acidosis was promptly corrected. This is in contrary to the documented literature which states that BCIS is a time-limited process^17^ and healthy hearts can recover within minutes, even from large embolic loads associated with cemented implantation. BCIS is reversible even in elderly, critically ill patients, if their hemodynamic stability is maintained by supportive therapy^18^. We maintained supportive therapy but could not salvage the patient.

Case 2 also had BCIS with severe hypotension and respiratory arrest. Endotracheal intubation and artificial ventilation was required and because of prompt resuscitation, patient recovered with out any residual sequel.

Case 3 had BCIS during shoulder replacement. She had hypotension but patient recovered immediately due to institution of fluid and vasopressor therapy. In literature search we did not come across any report of BCIS during shoulder replacement.

Case 4 had hypoxemia after knee arthroplasty without any hypotension and bradycardia. This patient required oxygen supplementation (initially 100% and then via venturi mask FiO_2_ 0.5) for 48 hours to maintain SpO_2_ more than 97%. Presence of tourniquet modified the time of events and limited the vasodilatory consequences of cement insertion, but it could not prevent embolization and BCIS^19^.

All 4 cases were females. At our centre 65% cases operated for joint replacement surgeries are females. There is more incidence of osteoporosis and osteoar-thritis in females but no relation of gender for occurrence of cement embolism.

Toxic effects of methylmethacrylate were considered the major cause of hemodynamic instability during arthroplasty surgery^1^,^14^. However, this hypothesis has not been supported by animal studies. Greater than 30 times the level of methylmethacrylate is required to create hemodynamic changes as seen in humans^1^,^14^,^20^.

Absorbed monomer temporarily lowers blood pressure after insertion of bone cement, there is little evidence indicating that monomer causes severe systemic reactions^21^. Orsini et al postulated that the pressurizing effect of bone cement or inert bone wax produces high intramedullary pressures^1^.

Hemodynamic effects of medullary fat embolism, rather than the toxic effects of the cement itself cause BCIS^17^,^22^,^23^. The endogenous cannabinoids, anandamide (ANA) and 2-arachidonylglycerol (2-AG) are reported to be strong vasodilators and play a role in the hypotension associated with BCIS^16^.

In all cases it was the effect of fat embolization due to cement insertion. Studies have ruled out direct toxic effect of cement monomer^1^,^14^,^20^.

Cement placement results in sealing and prosthesis insertion causes pressurization of intramedullary canal^1^. High intramedullary pressure (often>300mmHg) forces medullary fat into the blood vessels. This embolic load can cause systemic, life-threatening hypotension^1^,^17^,^23^–^25^, pulmonary hypertension^14^,^17^,^23^,^25^, increased central venous pressure^25^, pulmonary edema^14^, bronchoconstriction^14^, hypoxemia^1^,^14^,^23^,^25^,^27^, decreased EtCO_2_^24^,^27^, cardiac dysrhythmia^27^, cardiogenic shock^27^, cardiac arrest^27^, sudden death^27^, hypothermia^27^, thrombocytopenia^27^ etc. Less hemodynamic disturbances are seen in uncemented arthroplasty as lower intramedullary pressures, fewer and smaller emboli are produced^17^. These evidences support an embolic etiology as the mechanism for cardiac arrest during arthroplasty involving the use of cement^2^.

Venous embolization can be detected by transesophageal echocardiography (TEE) during cemented total hip arthroplasty^20^,^26^. TEE might have been useful in the management, but was not readily available to the operation theatres at the time these cases occurred and is not routinely used at this centre.

Anaesthetic management of the bone implantation syndrome is supportive. Administration of 100% inspired oxygen with control of airway, invasive hemodynamic monitoring, aggressive volume therapy and use of vasopressors is required^2^,^18^. Surgical modifications to prevent excessive cement pressurization are paramount in avoiding or minimizing BCIS as complete elimination of the embolic phenomenon is probably impossible^2^,^18^,^27^. Prophylactic use of antihistaminics or steroids for treatment of cement embolism could not be found in the literature search.

To conclude BCIS is a significant cause of morbidity and mortality in orthopedic surgery. High index of suspicion and close monitoring is required at the time of cement insertion for early clinical diagnosis. Surgical modifications, early and aggressive resuscitation with use of vasopressors and invasive haemodynamic monitoring are the key to the prevention of catastrophic outcome.

## References

[1] Orsini EC, Byrick RJ, Mullen JB, Kay JC, Waddell JP (1987). Cardiopulmonary function and pulmonary microemboli during arthroplasty using cemented or non-cemented components. The role of intramedullary pressure. J Bone Joint Surg Am.

[2] Patterson BM, Healey JH, Cornell CN, Sharrock NE (1991). Cardiac arrest during hip arthroplasty with a cemented long-stem component. A report of seven cases. J Bone Joint Surg Am.

[3] McLaughlin RE, DiFazio CA, Hakaka M, Abbott B, MacPhail JA, Mack WP, Sweet DE (1973). Blood clearance and acute pulmonary toxicity of methylmethacrylate in dogs after simulated arthroplasty and intravenous injection. J Bone Joint Surg Am.

[4] Murphy P, Edelist G, Byrick RJ, Kay JC, Mullen JB (1997). Relationship of fat embolism to haemodynamic and echocardiographic changes during cemented arthroplasty. Can J Anaesth.

[5] Duncan JA (1989). Intra-operative collapse or death related to the use of acrylic cement in hip surgery. Anaesthesia.

[6] Enneking FK (1995). Cardiac arrest during total knee replacement using a long-stem prosthesis. J Clin Anesth.

[7] Fallon KM, Fuller JG, Morley-Forster P (2001). Fat embolization and fatal cardiac arrest during hip arthroplasty with methylmethacrylate. Can J Anesth.

[8] Lafont ND, Kostucki WM, Marchand PH, Michaux MN, Boogaerts JG (1994). Embolism detected by transesophageal echocardiography during hip arthroplasty. Can J Anaesth.

[9] Motobe T, Hashiguchi T, Uchimura T, Yamakuchi M, Taniguchi N, Komiya S, Maruyama I (2004). Endogenous cannabinoids are candidates for lipid mediators of bone cement implantation syndrome. Shock.

[10] Cohen CA, Smith TC (1971). The intraoperative hazard of acrylic bone cement: report of a case. Anesthesiology.

[11] Frost PM (1970). Cardiac arrest and bone cement. Br Med J.

[12] Hyland J, Robins RH (1970). Cardiac arrest and bone cement. Br Med J.

[13] Schuh FT, Schuh SM, Viquera MG, Terry RN (1973). Circulatory changes following implantation of methylmethacrylate bone cement. Anesthesiology.

[14] Byrick RJ, Forbes D, Waddell JP (1986). A monitored cardio-vascular collapse during cemented total knee replacement. Anesthesiology.

[15] Fahmy NR, Chandler HP, Danylchuk K, Matta EB, Sunder N, Siliski JM (1990). Blood-gas and circulatory changes during total knee replacement Role of the intramedullary alignment rod. J Bone Joint Surg Am.

[16] Orsini EC, Richards RR, Mullen JM (1986). Fatal fat embolism during cemented total knee arthroplasty: a case report. Can J Surg.

[17] Byrick RJ (1997). Cement implantation syndrome: a time limited embolic phenomenon. Can J Anaesth.

[18] (2006). Pennsylvania Patients Safety reporting system. PA-PSRS Pat Saf Advis.

[19] Enneking FK (1995). Cardiac arrest during total knee replacement using a long-stem prosthesis. J Clin Anesth.

[20] Murphy P, Edelist G, Byrick RJ, Kay JC, Mullen JB (1997). Relationship of fat embolism to haemodynamic and echocardiographic changes during cemented arthro-plasty. Can J Anaesth.

[21] Kirwan WO (1973). Systemic phenomena and bone cement. Ir J Med Sci.

[22] Clark DI, Ahmed AB, Baxendale BR, Moran CG (2001). Cardiac output during hemiarthroplasty of the hip. A prospective, controlled trial of cemented and uncemented prostheses. J Bone Joint Surg Br.

[23] Jenkins K, Wake PJ (2002). Cement implantation syndrome. Anaesthesia.

[24] Lafont ND, Kostucki WM, Marchand PH, Michaux MN, Boogaerts JG (1994). Embolism detected by transoesophageal echocardiography during hip arthroplasty. Can J Anaesth.

[25] Parry G (2003). Sudden deaths during hip hemi-arthroplasty. Anaesthesia.

[26] Parvizi J, Holiday AD, Ereth MH, Lewallen (1999). Sudden death during primary hip arthroplasty. Clin Orthop Relat Res.

[27] Byrick RJ, Bell RS, Kay JC, Waddell JP, Mullen JB (1989). High-volume, high-pressure pulsatile lavage during cemented arthroplasty. J Bone Joint Surg Am.

